# Bread Wheat Quality: Some Physical, Chemical and Rheological Characteristics of Syrian and English Bread Wheat Samples

**DOI:** 10.3390/foods1010003

**Published:** 2012-11-22

**Authors:** Abboud Al-Saleh, Charles S. Brennan

**Affiliations:** 1 Food Science Department, Faculty of Agriculture, Al Furat University, Deir-Ezzor, Syria; Email: abbalsaleh@yahoo.co.uk; 2 Department of Wine, Food and Molecular Biosciences, Lincoln University, Lincoln 7647, Canterbury, New Zealand

**Keywords:** bread wheat, kernel characteristics, flour, dough, bread quality, sensory analysis

## Abstract

The relationships between breadmaking quality, kernel properties (physical and chemical), and dough rheology were investigated using flours from six genotypes of Syrian wheat lines, comprising both commercially grown cultivars and advanced breeding lines. Genotypes were grown in 2008/2009 season in irrigated plots in the Eastern part of Syria. Grain samples were evaluated for vitreousness, test weight, 1000-kernel weight and then milled and tested for protein content, ash, and water content. Dough rheology of the samples was studied by the determination of the mixing time, stability, weakness, resistance and the extensibility of the dough. Loaf baking quality was evaluated by the measurement of the specific weight, resilience and firmness in addition to the sensory analysis. A comparative study between the six Syrian wheat genotypes and two English flour samples was conducted. Significant differences were observed among Syrian genotypes in vitreousness (69.3%–95.0%), 1000-kernel weight (35.2–46.9 g) and the test weight (82.2–88.0 kg/hL). All samples exhibited high falling numbers (346 to 417 s for the Syrian samples and 285 and 305 s for the English flours). A significant positive correlation was exhibited between the protein content of the flour and its absorption of water (*r* = 0.84 **), as well as with the vitreousness of the kernel (*r* = 0.54 *). Protein content was also correlated with dough stability (*r* = 0.86 **), extensibility (*r* = 0.8 **), and negatively correlated with dough weakness (*r* = −0.69 **). Bread firmness and dough weakness were positively correlated (*r* = 0.66 **). Sensory analysis indicated Doumah-2 was the best appreciated whilst Doumah 40765 and 46055 were the least appreciated which may suggest their suitability for biscuit preparation rather than bread making.

## 1. Introduction

Bread wheat is an important crop worldwide especially in the Mediterranean basin. During the last ten years Syrian bread wheat production constituted more than half of Syria’s total wheat production [1]. This level of production can be explained by the importance of bread in the Syrian diet. Bread is considered the staple food for most people and consumed on a daily basis with two-layered bread in the most common form of bread in Syria and its neighbouring countries such as Lebanon, Jordan and Iraq. However, due to changes in dietary habits other forms of bread, such as Western style bread, can be found on the Syrian market and its consumption is increasing. The Western forms of bread are generally made in small bakeries using local flours; however no research has been carried out to explore the suitability of Syrian genotypes for the production of Western style bread. Current Syrian breeding programs are attempting to satisfy demand for bread making flours. For instance, researchers have tried to use the kernel physical traits (vitreousness, hardness, and specific weight), chemical characteristics of the flour (protein, ash, starch) and rheological properties of the dough (elasticity, resistance) to predict the quality of bread [2] or other cereal products [3,4].

The baking potential of wheat flours is influenced by many factors, most notably protein content [5,6,7,8]. Protein content is in turn influenced mainly by nitrogen fertilization, while the protein quality is determined primarily by the wheat genotype [3,4]. On the other hand, both the quality and the content of the wheat protein are affected by the climatic conditions during wheat maturation [9,10,11]. Vitreousness is considered to be related to the endosperm microstructure whereas hardness is suggested to influence the adhesion forces between starch granules and protein matrix [12]. Using election microscopy techniques and chemical analysis, a strong relationship between vitreousness and protein content was illustrated. Consequently, vitreousness can be used to predict the quality of kernel wheat end-uses [4,13]. Many studies investigating bread wheat baking performance have addressed protein properties, with particular emphasis on gluten strength. Differences in baking quality among cultivars have been related to differences in gluten composition, particularly to the high molecular weight glutenin subunits [7,14,15,16,17]. However, the separation of protein fractions, especially gliadins and glutenins, is still difficult due its dependency on the extraction conditions, which minimize the possibility to predict the baking quality in correlation with the flour characteristics [14,16].

In recent years, various researchers have attempted to avoid the baking tests by predicting bread quality through prediction models in which combination of measurements made from grain, flour and dough were used. For instance when trying to predict loaf volume, Miller [18] included glutenin quantity, gliadins percentage, flour colour grade, protein content, glutenin elastic modulus, farinograph water absorption, particle size index, moisture content, and the ratio of HMW to LMW glutenins in his equation for optimal flour content. Lee *et al.* [19] included in their prediction equation grain protein content, hardness index, mixograph water absorption and peak height, and break flour extraction and managed to achieve an *R*^2^ = 0.70 in terms of equation to product quality. Dowell *et al.* [20] combined grain, flour, and dough quality measurements into models to predict bread quality. They concluded that loaf volume and baking mix time, and water absorption could be predicted with *R*^2^ of 0.78–0.93. The three previous works cited indicate the difficulty in developing models to predict wheat baking performance, and confirms it is still necessary to perform baking tests in order to achieve a reliable evaluation.

The purpose of the present study was to investigate the major physical, chemical and rheological characteristics of some widely cultivated varieties in addition to some of those in the process of accreditation. The impact of these characteristics on the quality of the produced bread was also studied. For reliable evaluation of the ability of the Syrian genotypes for Western style bread preparation, a comparative study with some commonly used English flour was conducted. Consumer judgment is the most valuable tool in food quality assessment; therefore, a sensory analysis for bread quality evaluation was performed using a panel of assessors who regularly consume the product. 

## 2. Experimental Section

### 2.1. Materials

Six Syrian bread wheat genotypes, Sham-6, Sham-8, Bouhouth-8, Doumah-3, Doumah-40765 and Doumah-46055 were selected. These genotypes are recommended for sowing in irrigated areas. Doumah 40765 and Doumah 46055 genotypes are awaiting certification. Samples were provided from the Ministry of Agriculture, the General Commission for Scientific Agricultural Research, Deir Ezzor Research Station, which is located in the east of Syria. Genotypes were grown at the same location and under the same agro ecological conditions in the crop year 2009. The English flour samples were obtained from the commonly used flour in the market (Smith Flour Mills, Worksop, UK).

### 2.2. Physical Analyses

#### 2.2.1. Test Weight

Test weight was determined using the approved method of the American Association of Cereal Chemists 55-10 [21], and the results were reported in kg/hL. Damaged kernels were separated from the sound kernels for all the samples for the thousand kernel weight test, thousand grains were counted and weighed [3]. The degree of virtuousness was determined manually by sorting kernels, clean samples were used and 100 kernels were examined visually and separated into two groups, kernels free of starchy or speckled appearance called vitreous and those of starchy appearance named mealy [3,4]. Each sample was tested in triplicate. 

#### 2.2.2. Chemical Analyses

Water content of the kernels and the flour was determined by the approved AACC method 44-15 [21]. Protein content was conducted using the Kjedahl method and expressed using the conversion factor *N* × 5.7 [22]. Falling number was determined using the approved AACC method 56-81 [21]. Ash content was determined using the approved method 08-01 [21]. Results were expressed at a 14% water content basis and each sample was tested in triplicate. 

#### 2.2.3. Colour

The colour of the flour and the breads was assessed using a Datacolour Spectra (Datacolour International Lawrenceville, NJ, USA). In the Datacolour spectrophotometer SF600 Plus, the colour of a sample is denoted by the three dimensions, *L* *, *a* * and *b* *. The *L* *, *a* * and *b* * readings were treated using the software Colortools V3.1. The L * value gives a measure of the lightness of the product colour from 100 for perfect white to zero for black as the eye would evaluate it. The redness+/greenness− and the yellowness+/blueness− are denoted by the *a* * and *b* * values respectively. The colour of each sample was measured three times.

#### 2.2.4. Flour Yield

Flour production was achieved by cleaning the samples using sieves. Samples were tempered to 14% water content overnight (based on previous trial work, data not shown). Moistened samples were milled into flour using a Brabender Quadrumat Junior Experimental Mill (Brabender Co. Duisburg, Germany). Flour extraction was expressed on a total product basis; its rate was 72%.

#### 2.2.5. Bread Preparation

Bread preparation was conducted using a recipie and a protocol supplied by an industrial partner (not identified by request). The recipe of dough preparation was as shown in Table 1. The flour (Smith Flour Mills, Worksop, UK), the improver (Diamond British Arkady, UK) and the white shortening (Promaline, Vandemoortele, Hounslow, UK) were placed in the mixing bowl (Hobart A120, Hobart MFG Co., Troy, OH, USA). The dehydrated yeast (Craft Bake, DCL, UK) was added to the warm water and then added to the flour and mixed on speed 1 for 1 min followed by 10 min at speed 2. Then the other ingredients were added. The dough was divided into 300 g pieces rounded and left to rest under a cover for 10 min. The dough was moulded into a loaf shape and placed in tins. The tins were then put in the prover at 42 °C and 80% RH until the dough level reaches the top level of tin. Dough pieces were transferred to the oven and baked for 25 min at the temperature of 230 °C. The bread loaves were cooled and packed for testing. In total 10 breads were produced for analysis. 

**Table 1:** Formulation of breads.

Constituent	Quantity (g)	% of Total Weight
Flour	1500	58.55
Yeast	30	1.17
Salt	27	1.05
Diamond improver	30	1.17
White shortening	75	2.93
Water	900	35.13

#### 2.2.6. Bread Dough Characteristics

The Extensibility of dough was studied using a texture analyzer (TA-XT plus, Stable Micro Systems, Godalming, Surrey, UK) calibrated for a load cell of 30 kg. The Extensibility of dough was determined by the tensile test using the Kieffer rig (setting: pre-test speed, 2.0 mm/s; test speed, 3.3 mm/s; post test speed, 10.0 mm/s; distance, 75 mm; trigger force, auto-5 g; data rate acquisition, 200 point per second). More than 15 strips of dough were tested from each batch. Water absorption of flour was evaluated using the farinograph apparatus. Rheological properties of dough (development time, stability, mixing time, weakness and resilience) were determined according to the approved AACC method 54-21.01 [21].

#### 2.2.7. Bread Quality Evaluation

Bread firmness was determined using a texture analyzer (TA-XT Plus, Stable Micro Systems, Godalming, Surrey UK), calibrated for a load cell of 30 kg. Bread loaves were sliced mechanically into 12.5 mm slice thickness using Greaf bread slicing machine (Graef, Bradford, UK). Two slices were stacked together for each test, discarding two end slices of the loaf. Bread firmness was measured with a probe 25 mm in diameter and at 40% compression. Firmness was defined as the maximum force obtained during compression. Bread firmness and resilience (ability to recover from compression) were measured on three successive days using the method described as AACC Standard Method 74-09 [21]. Breads were stored in sealed plastic bags at 25 °C. 

##### 2.2.7.1. Loaf Volume

Loaf volume was measured by small seeds displacement method [23]. A container was used to measure the volume using small grains. Rapeseeds were poured into the container of known volume until the bottom was covered. The loaf was placed inside the container which was then filled to the top with more seeds. The extra rapeseeds, which equal the loaf volume, were measured in a graduated cylinder. The specific volume of the loaf was calculated using the following equation (1):

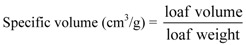
(1)


##### 2.2.7.2. Sensory Analysis

Sensory evaluation for bread was carried out under white light at room temperature in individual sensory booths at the Food Technology Laboratory, Hollings Faculty, MMU, Manchester, UK. The sensory laboratory conforms to ISO standard guidelines for the design of test rooms (ISO standard 8589, 1988) and ASTM’s physical requirements guidelines for sensory evaluation laboratories (ASTM 913, 1986). Bread was evaluated by generic descriptive analysis in accordance to Lawless and Heymann [24]. Ten experienced people were selected to assess the attributes of bread. Prior to sensory evaluation, the panellists were given the necessary training about the descriptors. For evaluation, samples were presented in 3-digit codes. Assessors evaluated colour, appearance, manual & oral texture, and flavour using a 10-point scale (Table 2). Data were recorded using the FIZZ computerised system version 1.20 (Biosystemes, Couternon, France). 

#### 2.2.8. Statistical Analyses

A total of 10 loaves of bread were made. All the experiments were replicated in triplicate unless otherwise stated. The coefficient of variability of all the tests was lower than 10%. Analysis of variance (ANOVA) and the linear regressions followed by Tukey’s test in addition to the correlations between factors were performed using SPSS 16.0 (SPSS Inc., Chicago, IL, USA). All the calculations were done at the significance level of *p* < 0.05. Correlation coefficients were run between the different variables using Microsoft Excel at the significance level of *p* < 0.05, 0.01, 0.005. 

**Table 2:** Descriptive vocabulary and definitions used by assessors to evaluate bread.

Attribute	Definition
*Appearance*	
Crust colour	Degree of colour darkness in the crust ranging from pale to dark brown
Crust colour	Degree of colour darkness in the crumb ranging from creamy to white
Crumb appearance	Degree of porosity and its uniformity from non uniform to uniform
*Odour*	
Yeasty	Odour associated with aromatic exchange from yeast fermentation
Grainy	An aromatic impression of cereal derived products like wheat, barley and corn
*Texture*	
Manual	Force required snapping sample by hand
Oral	Force required biting completely through sample placed between the molars
*Flavour*	
Sweet	Fundamental taste sensation of which sucrose is typical
Salt	Fundamental taste sensation elicited by sodium chloride
Sour	Fundamental taste sensation evoked by acids

## 3. Results and Discussion

### 3.1. Physical Properties

Physical characteristics such as vitreousness, kernel weight and test weight of the Syrian genotypes were determined to assess their contribution to the quality of the prepared bread. Vitreousness is an important factor in the determination of the quality of the wheat type because it reflects the texture of the endosperm and consequently the end use of the wheat [4,13,25]. Results showed that Bouhouth-8 had the highest percentage of vitreousness (95%) among all the Syrian genotypes (Table 3). There were no significant differences between the Doumah varieties, which varied from 69.3% to 76.6%, or the Sham genotypes. The 1000 kernel weight values showed a significant difference between most of the genotypes with values varying from 46 g (Doumah-2) to 35.2 g (Sham 6). Doumah’s genotypes yielded high 1000-kernel weight values (Table 3). Test weight values were generally high and showed significant differences between genotypes, for instance 82 kg/hL for Sham-6 to 88 kg/hL for Doumah-2. Doumah-40765 and Doumah-46055 (the latter are in the process of accreditation by the Syrian ministry of agriculture whereas Doumah-2 is already certified). The measurement of the water content showed slight differences among the genotypes. The low values reflect the drought of the environment during the period of harvesting in the eastern part of Syria where the genotypes were grown. The variations of values for the studied characteristics of the genotypes are likely to be the results of genotypic variation [26], as the agronomic conditions of the trial plots were as uniform as possible with regards to the field plot experiments.

**Table 3:** Selected physical characteristics of Syrian wheat samples.

Genotype	Vitreousness (%)	Kernel weight (g)	Test Weight (kg/hL)	Water content (%)
Doumah 2	71.33 ab	45.96 d	88.01 d	5.98 a
Doumah 40765	69.33 a	45.62 d	84.62 bc	5.94 a
Doumah 46055	76.67 abc	44.43 c	85.13 bc	6.01 a
Sham 6	81.67 bc	35.26 a	82.24 a	6.43 c
Sham 8	86.67 cd	37.31 b	85.80 c	6.28 bc
Bouhouth 8	95.00 d	36.02 a	84.20 b	6.15 ab

Values within columns with different letters are significantly different at *p* < 0.05.

Vitreousness was negatively correlated with the kernel weight and the test weight, the correlation values were *r* = −0.79 ** and *r* = −0.35 respectively. There was a significant positive correlation between the kernel weight and the test weight (*r* = 0.62 **) and a negative effect of the water content on the test weight of all the genotypes (*r* = −51 *) (Table 4).

**Table 4:** Correlation coefficients between physical characteristics of genotypes.

	Kernel Weight	Test Weight	Water Content
Vitreousness	−0.794 **	−0.347	0.544 *
Kernel weight		0.619 **	−0.831 **
Test weight			−0.509 *

* *p* < 0.05; **p < 0.01; *** *p* < 0.005.

### 3.2. Chemical Properties

A range of chemical properties of the Syrian and the English flour samples are presented in Table 5. The nutritional content of breads is related to the chemical composition of the bread, hence protein content and starch composition are of importance when considering the dietary impact of breads. Some significant differences were observed in the protein content of all the samples. For instance, among the Syrian samples Sham-6 had the highest protein content while Doumah-40765 had the lowest value. The falling number of the Syrian samples varied between 345 s for Sham-6 and 417 s for Doumah-2 which reflect a low α-amylase activity, while the English samples had lower falling number values around 300 s (Table 5). 

Protein content and vitreousness were correlated positively (*r* = 0.54 *), while a negative significant correlation between the protein content and the kernel weight was noticed (*r* = −0.8 **) (Table 4). A previous study on some Syrian durum wheat genotypes showed similar conclusions [26]. Results showed no correlation existed between the test weight and the protein content. A similar observation was mentioned by El-Khayat *et al.* [4]. The ash values for the Syrian samples did not show any significant difference among them and varied from 0.63% to 0.72% (on a dry basis), however there was a slight difference compared to the English samples (0.87% and 0.97%). Significant variations in the degree of water absorption of the flour samples were observed, varying from 56.3% for Doumah-40567 to 64% for the strong English sample. A correlation was noticed between the water absorption of the flour samples and its protein contents (*r* = 0.84 **) (Table 6). Concerning the colour of the flour, no significant difference was detected among most of the samples (Table 5). The colour of the flour samples were bright where L * values exceeded 92 for all of them except the English strong flour which is likely related to its high protein content (13.69%). Negative correlations were observed between the flour colour L * and the ash and protein with correlation values *r* of −0.62 ** and −0.78 ** respectively. This observation can be explained by the negative effect of the increase of both the protein and ash on the flour brightness. This conclusion can be enhanced by the negative correlation between the flour colour value L * and the water absorption. The redness values of the flour samples a * did not show significant difference, while the yellowness values b * varied from 8.57 for the weak English flour to 11.55 for Sham-8 which is due to more likely to the difference of pigments among samples, the negative significant correlation between b * values and ash values enhance the previous conclusion (*r* = −0.49 *).

**Table 5:** Chemical characteristics of wheat flour samples.

Genotype	Ash (g/100 g)	Protein (g/100 g)	Falling Number (sec)	Water Content (g/100 g)	Water Absorption (g/100 g)	Flour Colour *L*	Flour Colour *a*	Flour Colour *b*
Doumah 2	0.677 a	10.82 c	417.33 e	14.5 e	56.45 a	93.14 b	1.41 a	10.28 bcd
Doumah 40765	0.717 a	9.52 a	379.66 d	13.9 d	56.30 a	93.16 b	1.15 a	9.46 abc
Doumah 46055	0.690 a	10.06 b	351.66 c	13.0 b	59.40 c	92.52 b	1.56 a	10.46 cde
Sham 6	0.630 a	11.75 d	345.66 c	12.9 b	60.10 d	92.95 b	1.58 a	9.47 abc
Sham 8	0.723 a	11.02 c	372.33 d	13.1 b	61.40 e	92.05 b	1.75 a	11.55 e
Bouhouth 8	0.687 a	11.10 c	375.33 d	13.5 c	60.30 d	92.20 b	1.68 a	11.31 de
Strong English	0.973 b	13.69 e	285.66 a	11.1 a	64.05 f	89.93 a	1.10 a	9.18 ab
Weak English	0.873 b	9.77 ab	305.33 b	11.1 a	58.05 c	92.97 b	1.30 a	8.57 a

Values within columns with different letters are significantly different at *p* < 0.05.

### 3.3. Dough Properties

Flour samples showed considerable variations in the dough characteristics, where the mixing time varied from 1.5 min for the English weak samples to 3 min for Sham-6 and Bouhouth-8. The characteristics of the dough of both Doumah 40567 and 46055 were practically identical (Table 7). Dough samples also showed a wide range of dough stability, which varied from 1.5 min for Doumah 46055 to 8.4 min for the English strong sample. Doumah’s samples exhibited significant differences in weakness while Sham-6 had the least weakness value (52 BU) among the Syrian samples. Resistance values varied from 11.38 g to 39.34 g (Table 7) with Syrian dough samples showing less resistance than the English doughs. A negative correlation between dough mixing time and ash content was noticed (*r* = −0.69), while a positive correlation existed between the dough stability and the protein content of the samples (*r* = 0.86 **) and also with water absorption of the flour (*r* = 64 **) (Table 6). A negative correlation between dough weakness and protein content existed (*r* = −69 **), in addition to a logic negative correlation between dough weakness and its stability (*r* = −0.8). Dough extensibility was positively correlated with protein (*r* = 0.8 **) and also with the water absorption of the flour and the mixing time and resistance with r values of 0.69 **, 0.83 ** and 0.48 ** respectively. On the other hand, dough extensibility was negatively correlated with weakness (*r* = −0.78) (Table 6). Other researchers have reported that increased protein content generally increases dough extensibility due to the complex nature of the gliadin and glutenin proteins in forming a hydrated gluten matrix permitting controlled extension and elasticity of the dough [27,28,29,30,31]. 

**Table 6:** Correlation coefficients between characteristics of all samples.

	P	FN	WAbs	Flour L	Flour a	Flour b	Mixing Time	Stab	Weak	Res	Extn	Loaf Volume	Resil 0	Resil 1	Resil 2	Firm 0	Firm 1	Firmn 2
Ash	0.418 *	−0.750**	0.468	−0.623**	−0.149	−0.490 *	−0.688**	0.504 *	−0.375	0.809**	0.710**	0.055	−0.229	−0.179	−0.157	−0.439 *	−0.451*	−0.167
P		−0.440 *	0.837**	−0.775**	−0.035	−0.035	0.308	0.862**	−0.692**	0.192	0.804**	−0.096	0.499 *	0.657**	0.582**	−0.093	−0.112	−0.066
FN|			−0.611 *	0.557**	0.152	0.580**	0.481	−0.458	0.629**	−0.729**	−0.754**	−0.135	0.124	0.056	0.099	0.473 *	0.403	0.378
WAbs				−0.796**	−0.016	0.184	0.182	0.634**	−0.535*	0.190	0.685**	−0.059	0.328	0.345	0.420	−0.030	−0.028	0.132
Flour L					0.277	0.082	0.049	−0.703**	0.433	−0.354	−0.772**	−0.043	−0.334	−0.230	−0.267	0.152	0.135	0.009
Flour a						0.335	0.181	−0.134	−0.029	−0.149	−0.234	0.170	0.044	0.038	0.162	0.131	−0.136	0.180
Flour b							0.566 *	−0.134	0.346	−0.578**	−0.449*	−0.053	0.395	0.160	0.310	0.542**	0.358	0.609**
Mixingtime								0.132	−0.121	−0.571 *	−0.133	−0.034	0.450	0.733**	0.799**	0.238	0.446	0.192
Stab									−0.802**	0.397	0.832**	0.214	0.246	0.609 *	0.525*	−0.457	−0.404	−0.287
Weak										−0.490	−0.781**	−0.431	−0.037	−0.600 *	−0.532*	0.657**	0.473	0.585 *
Res											0.484**	0.281	−0.297	−0.201	−0.140	−0.585**	−0.517**	−0.430 *
Exten												0.066	0.147	0.308	0.267	−0.424*	−0.412 *	−0.294
Loafvolume													−0.362	0.072	0.158	−0.746**	−0.302	−0.625**
Resil 0														0.479 *	0.398	0.318	−0.017	0.132
Resil 1															0.793**	−0.112	−0.001	−0.295
Resil 2																−0.160	−0.071	−0.111
Firm 0																	0.668**	0.812**
Firm 1																		0.500*

P: protein; FN: falling number; WAbs: water absorption; Flour L: light colour of flour; Flour a: red colour of flour; Flour b: yellow colour of flour; Stab: Stabilisation; Res: resistivity; Extn: extensibility; Resil 0, 1, 2: Resilience of sliced bread at day 0, day 1, day 2; Firm 0, 1, 2: Firmness of sliced bread day 0, day 1, day 2; * *p* < 0.05, ** *p* < 0.01; *** *p* < 0.005.

**Table 7:** Characteristics of the dough samples.

Genotype	Mixing Time (min)	Stability (min)	Weakness (BU)	Resistance (g)	Extensibility (mm)
Doumah 2	2.5 c	4.0 cd	92.5 bc	15.57 c	12.62 a
Doumah 40765	2.0 b	2.5 ab	95.0 bc	11.38 a	12.22 a
Doumah 46055	2.0 b	1.5 a	105.0 c	12.35 ab	12.77 a
Sham 6	3.0 d	3.5 bc	52.5 ab	11.77 ab	25.89 c
Sham 8	2.5 c	2.5 b	105.0 c	13.55 b	12.52 a
Bouhouth 8	3.0 d	5.0 d	60.0 ab	18.50 d	17.07 ab
Strong	2.0 b	8.4 e	30.0 a	30.55 e	62.50 d
Weak	1.5 a	3.0 bc	67.5 abc	39.34 f	21.86 bc

Values within columns with different letters are significantly different at *p* < 0.05.

### 3.4. Bread Quality

#### 3.4.1. Functional Properties

The samples did not show significant differences in the specific volume of bread loaf which varied from 3.48 for Sham-6 to 3.73 for Bouhouth-8. All Syrian genotypes showed a similar ability to produce bread when compared to the English. The resilience 0 values did not show significant differences between samples except Doumah-40567 and the weak English flour sample which had low values compared to the others (Table 8). The resilience on day 2 (resilience-1) showed significant differences between samples where three groups can be distinguished. This difference between samples was magnified during the third day (resilience-3) values. A decrease of resilience values in relation with time are obvious (Table 8). The firmness 0 values showed three groups significantly different where Sham-8 had the highest value 965.9 g. The day-three firmness values (firmness-2) were not significantly different for all samples except for Sham-8 where its firmness value reached 1234.2 g (Table 8). The resilience of the bread has been shown previously to be related to the protein content of the flour and doughs [7,11], this can be supported by the positive correlations between these values observed in Table 6. 

**Table 8:** Characteristics of the bread samples.

Genotype	Specific Volume	Resilience 0 (g)	Resilience 1 (g)	Resilience 2 (g)	Firmness 0 (g)	Firmness 1 (g)	Firmness 2 (g)
Doumah-2	3.530 a	62.94 b	52.48 b	48.59 bcd	720.34 b	822.68 ab	961.55 a
Doumah-40765	3.630 a	45.46 a	45.46 a	41.44 a	590.11 ab	833.09 ab	899.62 a
Doumah-46055	3.565 a	64.06 b	44.68 a	42.66 ab	697.58 b	775.68 a	956.10 a
Sham-6	3.545 a	58.80 b	58.80 c	52.37 de	689.94 b	929.45 ab	852.70 a
Sham-8	3.485 a	56.92 b	46.07 a	45.07 abc	965.90 c	1072.26 b	1234.72 b
Bouhouth-8	3.735 a	58.06 b	58.06 bc	55.91 e	527.04 a	795.99 ab	837.85 a
Strong	3.600 a	61.51 b	54.82 bc	50.02 cde	503.50 a	670.50 a	854.97 a
Weak	3.640 a	46.09 a	42.32 a	41.20 a	498.04 a	714.63 a	798.37 a

Values within columns with different letters are significantly different at *p* < 0.05.

The daily results for bread resilience were positively correlated with the protein content of the samples with significant correlation values of 0.5 *, 0.7 ** and 0.6 ** respectively (Table 6). Another significant correlation with the flour mixing time was noticed, where correlation values were 0.45 *, 0.73 * and 0.79 **. Bread firmness during the three day time was positively correlated, where the correlation value (*r*) between firmness 1 and 2 was 0.67 **, and 0.81 ** with firmness 3, while the correlation value between firmness2 and 3 was 0.5 * (Table 6). A positive correlation was detected between firmness in its three stages and dough weakness with r values as follows: 0.66 ** for day-one firmness, 0.47 * for day two firmness and 0.59 * for day-three firmness, in addition to significant negative correlations with dough resistance where r values were −0.59 **, −0.52 **and −0.43 * for the three successive days (Table 6). The correlation values illustrate the effect of the protein content on the quality of the bread and these results are in general agreement with numerous studies highlighted the close relationship between bread-making quality of the wheat flour and the grain protein content [8,10,32,33].

#### 3.4.2. Sensory Analysis

The results of the sensory analysis of the bread samples are presented in Table 9. Although the results were generally not significantly different, the results revealed trends amongst the bread. For instance Doumah-40765 had the highest score for the colour of the crust (5.77) among all samples whereas the weak English sample had the lowest value (2.58) (Table 9). Similarly there was a trend concerning the colour of the crumb with Sham-8 preferred the most (5.97) while the strong English flour was the least appreciated (2.18). Doumah-2 had the best crumb appearance (7.19) while Doumah-40765 was the least appreciated (2.62). Concerning the odour of the bread (yeasty and grainy) and its taste (sweetness, saltiness and sourness) in addition to crumb hardness, no significant differences were noticed among samples (Table 9).

**Table 9:** Bread sensory analysis.

Genotype	Crust Colour	Crumb Colour	Crumb Appear	Odour Yeasty	Odour Grainy	Crumb Hardness	Crumb Adhesion	Sweetness	Saltiness	Sourness	Bread Overall
Doumah 2	5.18 b	5.41 b	7.19 b	4.02 a	3.85 a	6.84 a	6.67 b	2.58 a	3.51 a	3.61 a	6.47 b
Doumah 40765	5.77 b	4.21 ab	2.62 a	4.70 ab	4.68 a	4.25 a	4.48 ab	2.88 a	2.84 a	3.65 a	2.91 a
Doumah 46055	4.89 b	4.33 ab	4.24 ab	5.15 ab	4.17 a	3.75 a	5.27 ab	2.69 a	3.33 a	4.08 a	3.63 ab
Sham 6	4.94 b	5.97 b	4.70 ab	3.84 a	4.69 a	5.29 a	5.85 ab	3.16 a	3.32 a	3.50 a	5.70 ab
Sham 8	4.35 b	3.57 ab	5.53 ab	6.30 b	4.18 a	4.39 a	5.87 ab	3.78 a	3.46 a	3.35 a	4.45 ab
Bouhouth 8	5.66 b	4.83 b	7.13 b	4.65 ab	4.44 a	6.12 a	5.13 ab	2.62 a	3.61 a	3.74 a	5.55 ab
Strong	5.56 b	2.18 a	5.33 ab	5.43 ab	4.59 a	5.73 a	5.06 ab	3.43 a	3.06 a	3.53 a	4.65 ab
Weak	2.58 a	4.12 ab	6.01 b	6.29 b	4.11 a	6.42 a	2.61 a	2.98 a	3.50 a	4.65 a	4.35 ab

Values within columns with different letters are significantly different at *p* < 0.05.

As can be seen in Table 8, the crumb of Doumah-2 was appreciated the most by the assessors in terms of crumb adhesion in the mouth (6.67), on the other hand, the weak English sample was the most sticky when chewed between molars which gains a poor response from the assessors (2.61). The other samples gained similar response from the panellists, where no significant differences were observed (Table 9). The results of bread overall rating revealed three distinct groups significantly different, where Doumah-2 was the most appreciated (6.47). The results for the sensory properties of the bread provide interesting preliminary findings.

## 4. Conclusions

The results of our research illustrate variations between Syrian genotypes in vitreousness, kernel weight and test weight; differences were also noticed in protein content, falling number, flour water absorption and the colour of the flour in all Syrian and English samples. Experimental lines, Doumah-40765 and Doumah-46055 exhibited reduced vitreousness and protein content compared to any of the other genotypes. Samples showed clear differences in dough and bread characteristics (resilience and firmness). The differences in physical, chemical and rheological characteristics between samples did not give significant variations in specific loaf volume, but those differences affected the quality of the loaf. Bread sensory analysis clearly demonstrated the effect of the kernel physical characteristics, flour chemical traits and the dough rheology on bread quality. Colour, appearance and texture were the major factor in bread evaluation. Results proved conclusively the validity of the Syrian genotypes for Western style bread making which is not one of the mostly commonly produced bread on the Syrian market. The Syrian variety Doumah-2 was the most appreciated among all samples, while Doumah 40765 and 46055 were the least appreciated by the assessors which may suggest their suitability for biscuit preparation rather than bread making. Correlation analyses confirmed the importance of the vitreousness, protein content and the rheological traits of dough on the quality of bread especially the resilience and firmness. It is common in breeding programs to assess the suitability of grain solely on the properties of the raw material. In this study, sensory analysis was prioritized to assess bread quality rather than relying on judging the quality of the final product merely through quantitative evaluation of kernel and flour characteristics. 
